# Development of an online patient decision aid for kidney failure treatment modality decisions

**DOI:** 10.1186/s12882-022-02853-0

**Published:** 2022-07-06

**Authors:** Noel Engels, Paul B. van der Nat, Jet W. Ankersmid, Janine C. M. Prick, Ellen Parent, Regina The, Asako Takahashi, Hans A. J. Bart, Cornelia F. van Uden-Kraan, Anne M. Stiggelbout, Willem J. W. Bos, Marinus A. van den Dorpel

**Affiliations:** 1 Department of Shared Decision-Making and Value-Based Health Care, Santeon, Utrecht, the Netherlands; 2 Department of Internal Medicine, Maasstad Hospital, Rotterdam, the Netherlands; 3 Department of Internal Medicine, Leiden University Medical Centre, Leiden, the Netherlands; 4 Department of Value-Based Health Care, St. Antonius Hospital, Nieuwegein, the Netherlands; 5 IQ Healthcare, Radboud University Medical Centre, Nijmegen, the Netherlands; 6 Department of Neurology, OLVG, Amsterdam, the Netherlands; 7 Department of Quality and Improvement, Maasstad Hospital, Rotterdam, the Netherlands; 8 Department of Development, ZorgKeuzeLab, Delft, the Netherlands; 9 Department of Shared Decision-Making and Value-Based Health Care, Nierpatiënten Vereniging Nederland, Bussum, the Netherlands; 10 Department of Biomedical Data Sciences, Leiden University Medical Centre, Leiden, the Netherlands; 11 Department of Internal Medicine, St. Antonius Hospital, Nieuwegein, the Netherlands

**Keywords:** Chronic kidney disease, End-stage kidney disease, Kidney replacement therapy, Conservative care management, Shared decision-making, Patient decision aid

## Abstract

**Background:**

Patient decision aids (PtDAs) support patients and clinicians in shared decision-making (SDM). Real-world outcome information may improve patients’ risk perception, and help patients make decisions congruent with their expectations and values. Our aim was to develop an online PtDA to support kidney failure treatment modality decision-making, that: 1) provides patients with real-world outcome information, and 2) facilitates SDM in clinical practice.

**Methods:**

The International Patient Decision Aids Standards (IPDAS) development process model was complemented with a user-centred and convergent mixed-methods approach. Rapid prototyping was used to develop the PtDA with a multidisciplinary steering group in an iterative process of co-creation. The results of an exploratory evidence review and a needs-assessment among patients, caregivers, and clinicians were used to develop the PtDA. Seven Dutch teaching hospitals and two national Dutch outcome registries provided real-world data on selected outcomes for all kidney failure treatment modalities. Alpha and beta testing were performed to assess the prototype and finalise development. An implementation strategy was developed to guide implementation of the PtDA in clinical practice.

**Results:**

The ‘Kidney Failure Decision Aid’ consists of three components designed to help patients and clinicians engage in SDM: 1) a paper hand-out sheet, 2) an interactive website, and 3) a personal summary sheet. A ‘patients-like-me’ infographic was developed to visualise survival probabilities for each treatment modality on the website. Other treatment outcomes were incorporated as event rates (e.g. hospitalisation rates) or explained in text (e.g. the flexibility of each treatment modality). No major revisions were needed after alpha and beta testing. During beta testing, some patients ignored the survival probabilities because they considered these too confronting. Nonetheless, patients agreed that every patient has the right to choose whether they want to view this information. Patients and clinicians believed that the PtDA would help patients make informed decisions, and that it would support values- and preferences-based decision-making. Implementation of the PtDA has started in October 2020.

**Conclusions:**

The ‘Kidney Failure Decision Aid’ was designed to facilitate SDM in clinical practice and contains real-world outcome information on all kidney failure treatment modalities. It is currently being investigated for its effects on SDM in a clinical trial.

**Supplementary Information:**

The online version contains supplementary material available at 10.1186/s12882-022-02853-0.

## Introduction

Guidelines on the management of chronic kidney disease (CKD) emphasize the importance of kidney failure treatment modality education and decisional support as patients progress to the more advanced stages of CKD [1–3]. Shared decision-making (SDM) is recommended to support patients that have to make treatment modality decisions [3–5]. In addition, a recent study showed that a majority of patients prefer a SDM-approach when they have to make this decision [6].

Shared decision-making requires that patients and clinicians proactively engage in a collaborative decision-making process [7–9]. This process should be characterized by deliberation, during which patients become aware of their choice, understand all of their options, and get to consider what matters most to them. A three-step framework to help guide this process in clinical practice has been described [9]. This framework includes: 1) team talk, 2) option talk, and 3) decision talk. It also includes the use of decision support interventions (DSIs), such as patient decision aids (PtDAs), to further help patients and clinicians engage in SDM [9].

Patient decision aids are evidence-based tools that: 1) address a specific health-related decision, 2) provide patients with information on all of their options, and 3) guide them in decision-making through values-clarification and preferences-elicitation [10–12]. We previously identified 29 PtDAs developed to support patients with advanced CKD in treatment modality decision-making [13]. From the 27 PtDAs selected for detailed assessment, just about half qualified as PtDAs according to the International Patient Decision Aids Standards (IPDAS) collaboration minimum standards criteria [14]. In addition, about two-thirds were developed with the input of patients, the importance of which has been previously addressed [15]. End-users have a key role in the design of PtDAs, and involving them in the developmental phase facilitates implementation by addressing barriers to using PtDAs in clinical practice. Furthermore, patients and clinicians often value different treatment outcomes [16]. Therefore, patients should be involved to ensure patient-relevant outcomes are incorporated in PtDAs. This may improve their risk perception and help them make decisions congruent with their expectations and values [11].

Real-world outcome information is increasingly promoted to support decision-making based on local data rather than international data that do not match patient specific situations and risk being outdated or incomplete [17]. A recent study performed for the European Commission identified 192 initiatives investigating the use of real-world outcome information, 60 of which focused primarily on its use for decision-making [18]. In the Netherlands, the Dutch ministry of Health, Welfare and Sport has recently invested €70 million in a national campaign to encourage outcomes-based decision-making in clinical practice [19]. As part of this campaign, our objective was to develop an online PtDA for kidney failure treatment modality decisions that: 1) provides patients with real-world outcome information, and 2) facilitates SDM in clinical practice. In doing so, we: 1) evaluated which treatment outcomes were considered useful by patients to support these decisions, and 2) involved key stakeholders in its development so as to develop a PtDA that meets the needs and preferences of end-users. In this article, we report our findings and describe the development of the ‘Kidney Failure Decision Aid’.

## Materials and methods

### Study design and setting

The IPDAS development process model [15] (shown in Supplementary Material S1) was complemented with a user-centred and convergent mixed-methods approach [20] to develop the ‘Kidney Failure Decision Aid’.

First, a multidisciplinary steering group of key stakeholders was assembled, after which the steering group determined the target audience, scope, purpose, and general format of the PtDA. The steering group subsequently used the results of a previously performed scoping review [13] to guide an exploratory evidence review, and to determine how the PtDA could be integrated in established care pathways without interfering with routine procedures.

Afterwards, four steering group members (EP, RT, AT, NE) conducted a needs-assessment according to the Ottawa Hospital Research Institute’s (OHRI) guidelines to ascertain end-user preferences regarding the content and format of the PtDA [21]. Patients and clinicians were approached to participate in online surveys and focus groups to explore their experiences, needs, and preferences regarding (shown in Supplementary Material S2): 1) treatment modality education and decision-making, 2) SDM, 3) online PtDAs, and 4) outcome information. Patients were presented with 15 outcomes derived from the International Consortium for Health Outcome Measurement (ICHOM) [22] and the Standardised Outcomes in Nephrology (SONG) [23, 24] initiative, and asked to indicate whether they considered these outcomes useful for treatment modality decision-making. Outcomes were ranked from most to least useful based on the proportion of patients that considered each outcome useful. Outcomes were considered useful, or very useful, if more than 60% or 70% of patients voted for them, respectively.

The steering group subsequently developed the PtDA in an iterative process of co-creation, during which it collaborated with the Dutch kidney patient association (NVN). Real-world outcome information was obtained through a partnership with seven collaborating Dutch teaching hospitals (the Santeon hospitals) [25] and national Dutch registries that manage dialysis and kidney transplantation outcomes. Rapid prototyping was employed to develop low-fidelity versions of the PtDA. When the prototype was considered ‘complete’, a high-fidelity version was developed and used for alpha and beta testing. Alpha testing is defined as: “obtaining direct feedback from ‘typical’ users during the developmental process. This may include members of the steering group and others involved in the developmental process.” [15]. Beta testing is defined as: “testing with patients and clinicians external to the developmental process, where possible in ‘real-world’ settings, to assess feasibility.” [15]. During the alpha testing, four steering group members (JH, EP, RT, NE) assessed whether the prototype: 1) met the identified needs and preferences of end-users (patients and clinicians), 2) contained information on the treatment outcomes that patients considered useful, or very useful, for treatment modality decision-making, and 3) met all IPDAS minimum standards criteria and additional IPDAS criteria for internet based PtDAs [12, 14]. Three steering group members (RT, AT, NE) subsequently performed the beta testing, during which patients and clinicians were asked to assess the contents and format of the prototype during think-aloud sessions. The results were subsequently used by the steering group to optimize the prototype for clinical practice, and finalise development.

Finally, the steering group developed an implementation strategy based on an approach that focuses on removing barriers for the implementation of PtDAs [26, 27].

### Participants, recruitment and informed consent

Adult patients with stage four to five CKD [1] and clinicians involved in kidney failure treatment modality education and decisional support were eligible to participate in the needs-assessment and beta testing of the prototype.

Participants were recruited by means of purposive sampling through an online platform of the Dutch kidney patient association and in the Santeon hospitals that are geographically spread out over the Netherlands. Patients were encouraged to bring caregivers to the focus groups.

The appropriate national and institutional regulatory authorities and ethics committees approved this study (registration no. W19.138). All participants provided written informed consent.

### Data analysis

Descriptive statistics were used to describe the demographic and clinical characteristics of the study participants, and to describe all other quantitative data obtained during the developmental process.

Continuous data are expressed as a mean with standard deviation (SD). Categorical data are expressed as frequencies (%) unless otherwise stated. Kaplan–Meier survival analysis was used to calculate survival and hospitalisation probabilities for each kidney failure treatment modality.

All focus groups and think-aloud sessions were audio-recorded and transcribed verbatim. Two independent coders (NE, JCMP) categorized the focus group data according to the topics discussed. The data were subsequently coded with an open coding approach, after which inductive thematic analysis [28] was used to identify participants’ needs and preferences regarding: 1) treatment modality education and decision-making, 2) SDM, 3) online PtDAs, and 4) outcome information. The consolidated criteria for Reporting Qualitative research (COREQ) checklist [29] was used as a framework to report the qualitative data.

All qualitative and quantitative data were analysed with ATLAS.ti (version 8) and IBM SPSS Statistics (version 26).

## Results

### The multidisciplinary steering group

The steering group consisted of 16 members: seven nephrologists, two patient representatives, one patient advocate of the Dutch kidney patient association, one nurse practitioner, one social worker, one researcher and nephrology resident, one VBHC professor, one health-policy and change-management specialist who was the project leader, and one communication scientist who was the project manager.

**Fig. 1:**
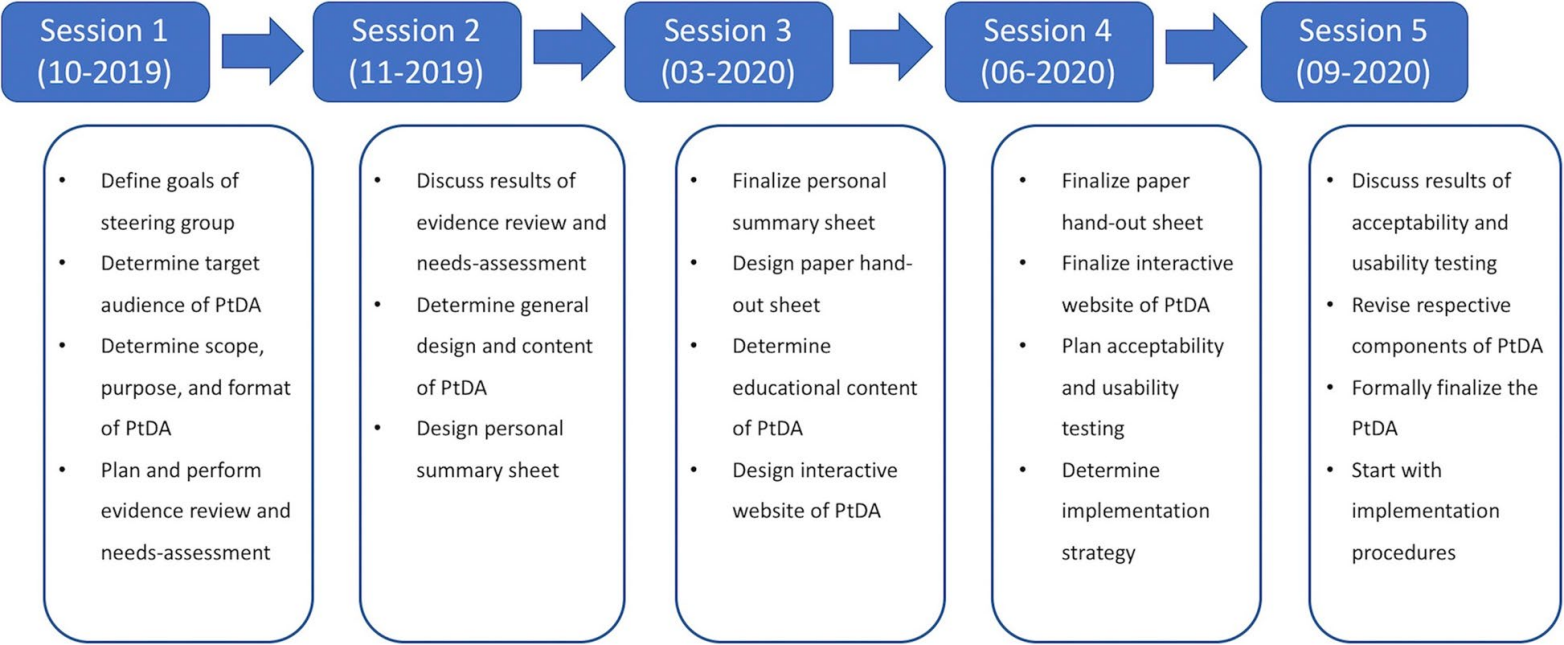
Overview of PtDA development. *PtDA* *=* *Patient decision aid*

The steering group convened in two live, and later due to the COVID-19 pandemic, three hybrid meetings between October 2019 and September 2020 to develop the PtDA (shown in Fig. 1).

The developmental process was facilitated by the general director and a user-experience specialist of ZorgKeuzeLab, a company specialized in PtDA development.

### Audience, scope, purpose and format

Patients with stage four and five CKD were selected as the target audience for the ‘Kidney failure Decision Aid’.

The steering group agreed that all patients should be able to use the PtDA, regardless of which treatment modalities they qualify for. Therefore, it was decided that the PtDA should contain information on every kidney failure treatment modality, and that patients should be instructed on what chapters they should read when it is handed out.

The steering group wanted the PtDA to focus on helping patients make values- and preferences-based decisions through the principles of SDM. Moreover, the incorporation of real-world outcome-information and values-clarification and preferences-elicitation exercises were considered essential to help patients make decisions that match their expectations. All members agreed that the PtDA should provide patients with all the necessary information they need on one platform, including information on common fears (e.g. phobia of needles) and misconceptions (e.g. switching or stopping treatment is not possible) regarding kidney replacement therapy (KRT). The group aimed to develop a PtDA that could be used throughout the Netherlands, and set out to collaborate with key stakeholders and educational services already frequently used to facilitate its uptake in clinical practice.

It was decided to develop an online PtDA that combined both printed and digital elements. Printed elements were considered useful to physically hand-out the PtDA and facilitate the SDM process in clinical practice. Digital elements, such as websites, are customizable to user preferences and allow for the integration of interactive content, such as: questions and exercises, infographics, and videos. Websites also offer the possibility of real-time feedback to track implementation and user fidelity.

### Exploratory evidence review

**Fig. 2:**
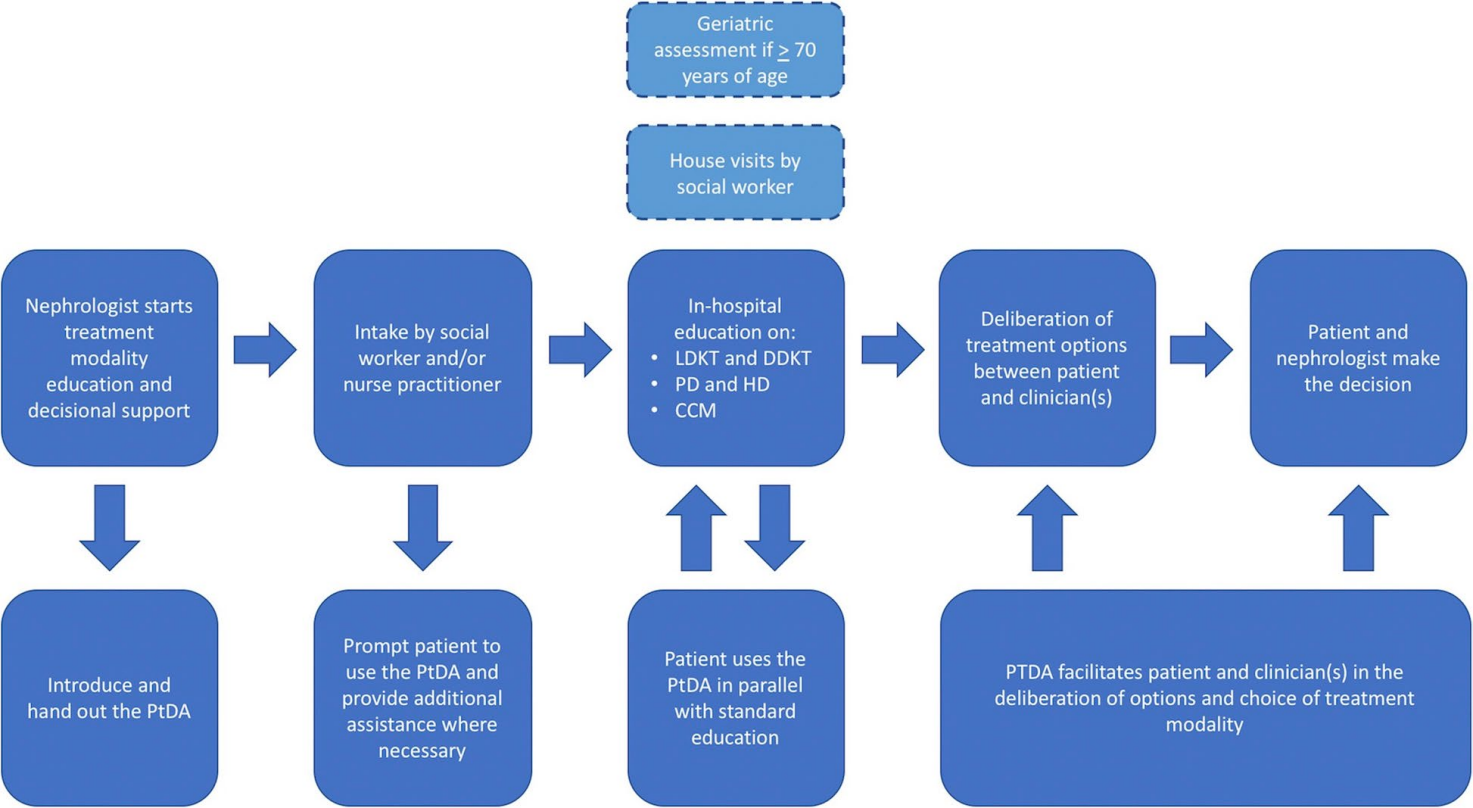
Overview of PtDA integration in Dutch advanced CKD care pathway. *PtDA =* *Patient decision aid*, *LDKT* = *Living donation kidney transplantation*, *DDKT = Deceased donation kidney transplantation*, *PD* = *Peritoneal dialysis*, *HD* = *Haemodialysis*, *CCM*  = *Conservative care management*

The evidence review revealed that a minority of PtDAs are implemented in clinical practice, and that new PtDAs should be streamlined with existing care pathways to facilitate implementation. Therefore, the steering group first considered how the PtDA could be successfully integrated in the Dutch advanced CKD care pathway while simultaneously facilitating SDM in clinical practice (shown in Fig. 2). In the Netherlands, nephrologists generally refer patients for treatment modality education and decisional support when their estimated glomerular filtration rate (eGFR) reaches 20 mL/min/1.73m^2^. This is where the SDM-process starts and the nephrologist should hand out the PtDA to the patient, as it can be utilized to support the first steps in this process (team talk and option talk). Afterwards, patients usually have an intake appointment with a social worker or nurse practitioner, which generally serves to: 1) further inform patients about the care pathway and its purpose, and 2) provide the nephrologist with additional information about a patient’s physical and psychosocial situation. For example, nurse practitioners will perform a geriatric assessment (to assess frailty) when patients are ≥ 70 years of age. In addition, social workers will conduct house visits (if patients agree to it) to: 1) assess whether a patient’s home is suitable for home-dialysis modalities, and 2) help patients discuss kidney transplantation options with their friends and family. The intake appointment can also be used to prompt patients to use the PtDA, and help them use it when necessary. Afterwards, patients usually have multiple in-hospital appointments with registered nurses for in-person education on the treatment modalities they qualify for. The contents of the PtDA should complement the education already provided by healthcare institutions, allowing patients to seamlessly use it as an additional source for education and deliberation in parallel with standard educational services. Finally, patients return to their nephrologist to discuss their options and make a treatment modality decision. This where the PtDA can be used to support patients and clinicians in the final step of the SDM process (decision talk).

### Needs-assessment: online surveys

A total of 125 patients (28%) and 42 clinicians (50%) responded to the online surveys. A survey completion rate of 98% and 76% was achieved among patients and clinicians.

Patients had a mean age of 59 years and half (53%) were male (shown in Supplementary Material S3). Clinicians had a mean age of 48 years and about two-thirds (69%) were female (shown in Supplementary Material S3). Patients are generally educated on their treatment options during outpatient consultations with nephrologists (97%), registered nurses (91%), and social workers (91%) (shown in Supplementary Material S4). Half of these clinicians (49%) use proprietary printed materials (49%), while about two-thirds (70%) use third-party printed materials. Clinicians also use educational websites, most of which use nierwijzer.nl (64%) and nieren.nl (58%) (shown in Supplementary Material S4). About two-thirds (70%) of clinicians reported being satisfied with the educational services they provide, and agreed (69%) that patients receive sufficient information to make treatment modality decisions (shown in Supplementary Material S4). In addition, the majority agreed (75%) or completely agreed (19%) that the information accessible to patients is reliable (shown in Supplementary Material S4). About half (43%) of the patients agreed that reliable information was easy to find, and half (52%) felt that the information supported them enough to make a treatment modality decision (shown in Supplementary Material S5). Three-quarters (78%) of the patients felt they had enough knowledge regarding their treatment options when they had to make their treatment modality decision (shown in Supplementary Material S5). Nevertheless, both patients and clinicians still considered an online PtDA to be helpful (39% and 66% respectively), or very helpful (19% and 19%) for treatment modality decision-making (shown in Table 1). A smaller proportion of patients than clinicians wished to have stories and experiences of peer-patients (35% vs. 72%), patient-reported outcomes (48% vs. 78%), or information on the effects of treatment modalities on social functioning and personal life (68% vs. 97%) in the PtDA (shown in Table 1).

**Table 1:** Patients’ and clinicians’ preferences regarding the use and content of an online PtDA to support kidney failure treatment modality decisions

Questions / statements	Patients	Clinicians
An online PtDA to help patients make treatment modality decision for kidney failure seems*	Very unhelpful = 6 (5%) Unhelpful = 10 (8%) Neutral = 36 (29%) Helpful = 49 (39%) Very helpful = 24 (19%)	Very unhelpful = 0 (0%) Unhelpful = 0 (0%) Neutral = 5 (16%) Helpful = 21 (66%) Very helpful = 6 (19%)
A detailed description of all treatment modalities for kidney failure should be included in the PtDA*	Yes = 94 (75%) No = 29 (23%) I don’t know = 2 (2%)	Yes = 28 (88%) No = 4 (12% I don’t know = 0 (0%)
Information regarding the pros and cons of each treatment modality should be included in the PtDA*	Yes = 107 (85%) No = 16 (13%) I don’t know = 2 (2%)	Yes = 28 (88%) No = 4 (12%) I don’t know = 0 (0%)
Stories and experiences of peer patients on the treatment modalities should be included in the PtDA*	Yes = 44 (35%) No = 79 (63%) I don’t know = 2 (2%)	Yes = 23 (72%) No = 9 (28%) I don’t know = 0 (0%)
Medical outcome information* (such as complication rates, hospitalisation) should be included in the PtDA	Yes = 79 (63%) No = 44 (35%) I don’t know = 2 (2%)	Yes = 23 (72%) No = 9 (28%) I don’t know = 0 (0%)
Patient reported outcome information* (such as pain, fatigue, physical functioning) should be included in the PtDA	Yes = 60 (48%) No = 63 (50%) I don’t know = 2 (2%)	Yes = 25 (78%) No = 7 (22%) I don’t know = 0 (0%)
Effects of the treatment modalities on social functioning and personal life* (such as being able to work, travel or do hobbies) should be included in the PtDA	Yes = 85 (68%) No = 38 (30%) I don’t know = 2 (2%)	Yes = 31 (97%) No = 1 (3%) I don’t know = 0 (0%)

*PtDA* = *Patient decision aid.* *Note: this is a translation from Dutch to English

Most patients considered the flexibility of each treatment modality (74%), the survival of each treatment modality after treatment initiation (74%), and the effect of each treatment modality on the residual kidney function over time (71%) useful for kidney failure treatment modality decision-making (shown in Table 2). The least number of patients (42%) considered patient-reported levels of depression on each treatment modality useful for this purpose.

**Table 2:** The usability of treatment outcomes for kidney failure treatment modality decisions according to patients

Ranked usability of treatment outcomes for treatment modality decision making according to patients 1 = highest, 15 = lowest	Results
1.The flexibility of each treatment modality*	Yes = 93 (74%) No = 20 (16%) I don’t know = 12 (10%)
2.The survival of each treatment modality after treatment initiation*	Yes = 92 (74%) No = 11 (9%) I don’t know = 21 (17%)
3.The effect of each treatment modality on the residual kidney function over time*	Yes = 89 (71%) No = 19 (15%) I don’t know = 17 (14%)
4.Patient reported levels of physical functioning on each treatment modality*	Yes = 82 (66%) No = 30 (24%) I don’t know = 13 (10%)
5.The effects of each treatment modality on social functioning*	Yes = 81 (65%) No = 31 (25%) I don’t know = 12 (10%)
6.Patient survival on each treatment modality*	Yes = 81 (65%) No = 27 (22%) I don’t know = 17 (13%)
7.The effects of each treatment modality on personal life*	Yes = 78 (63%) No = 33 (27%) I don’t know = 13 (10%)
8.Complication rates related to immunosuppressive drugs after transplantation*	Yes = 76 (61%) No = 34 (27%) I don’t know = 15 (12%)
9.Hospitalisation rates for each treatment modality after initiation*	Yes = 74 (60%) No = 36 (29%) I don’t know = 14 (11%)
10.Event rates for cardiovascular complications on each treatment modality*	Yes = 73 (58%) No = 29 (24%) I don’t know = 23 (18%)
11.Patient reported levels of pain on each treatment modality*	Yes = 67 (54%) No = 43 (35%) I don’t know = 14 (11%)
12.Vascular access survival in HD*	Yes = 67 (54%) No = 35 (28%) I don’t know = 23 (18%)
13.Patient reported levels of fatigue on each treatment modality*	Yes = 66 (53%) No = 40 (32%) I don’t know = 19 (15%)
14.PD peritonitis rates*	Yes = 64 (52%) No = 41 (33%) I don’t know = 18 (15%)
15.Patient reported levels of depression on each treatment modality*	Yes = 54 (43%) No = 54 (43%) I don’t know = 17 (14%)

*HD =* *Haemodialysis, PD* = *Peritoneal dialysis.* *Note: this is a translation from Dutch to English

### Needs-assessment: focus groups

Three focus groups were conducted. One with seven patients and one caregiver, and two with nine clinicians.

Patients and the caregiver had a mean age of 51 years and more than half (63%) was male (shown in Supplementary Material S6). Clinicians had a mean age of 52 years and three-quarters (79%) were female (shown in Supplementary Material S6).

A total of 15 needs and preferences of patients and clinicians regarding education and decision-making, SDM, an online PtDA, and the use of outcome information were identified in the data (shown in Table 3). Illustrative quotations supporting these needs and preferences have been extracted from the data (shown in Supplementary Material S7).

### Outcome information

**Fig. 3:**
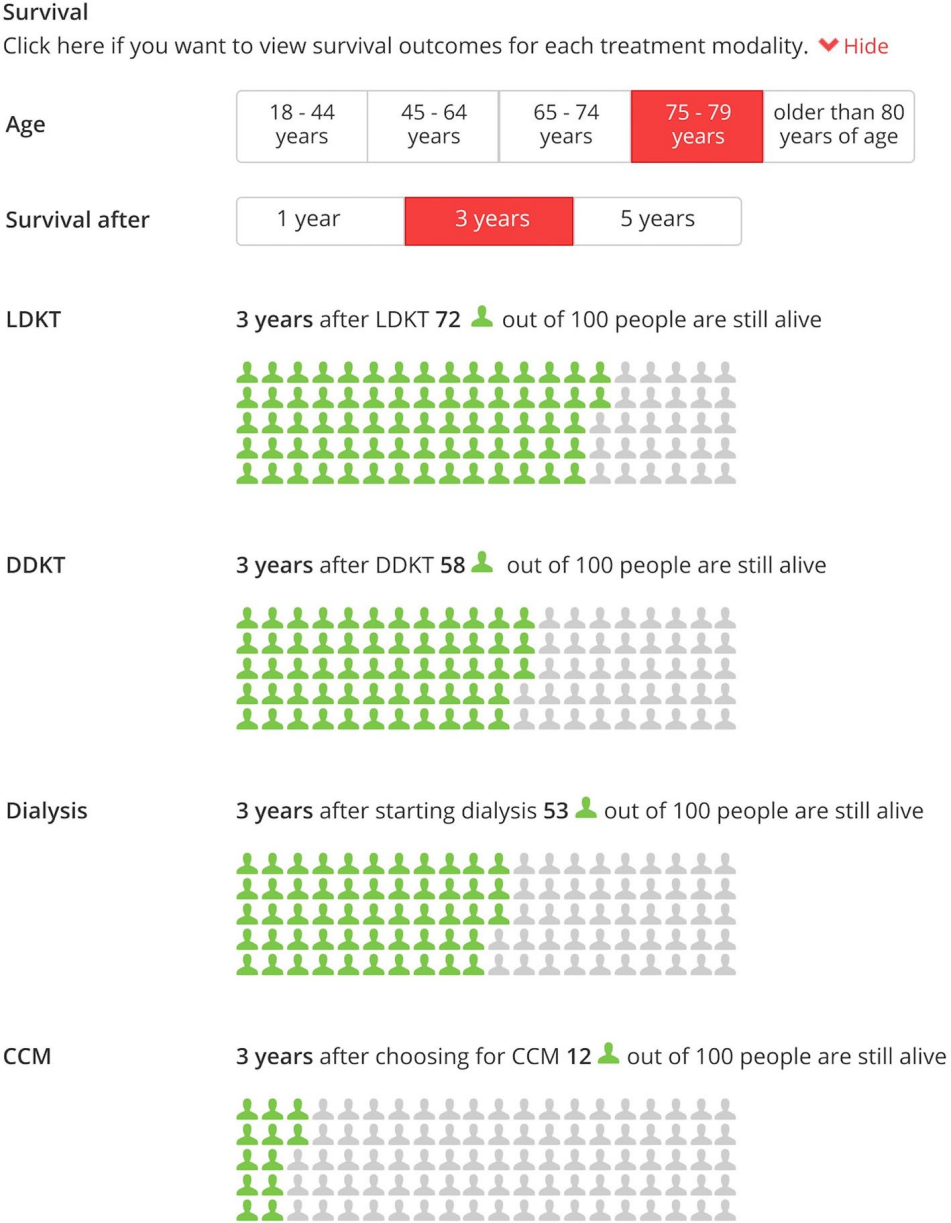
Survival probabilities in the ‘Kidney Failure Decision Aid’. *LDKT*  Living donation kidney transplantation, *DDKT*  Deceased donation kidney transplantation, *CCM*  Conservative care management. *Note: this is a translation from Dutch to English

Retrospective cohort data of 19.048 patients that were treated for kidney failure in Dutch hospitals between 2004 and 2020 were used to calculate survival probabilities and hospitalisation rates for each treatment modality. Kidney transplantation survival data were stratified in living donation kidney transplantation (LDKT) and deceased donation kidney transplantation (DDKT) groups. Dialysis survival data were pooled due to insignificant differences in survival between peritoneal dialysis (PD) and haemodialysis (HD). One- and three-year survival probabilities for dialysis and kidney transplantation were calculated with data from 2015 to 2020. Five-year survival probabilities for dialysis and kidney transplantation were calculated with data from 2010 to 2020. Annual hospitalisation rates due to treatment complications were calculated with data from 2015 to 2020. Data from 2004 and onwards were used for the calculation of survival probabilities and hospitalisation rates for conservative care management (CCM) due to the limited number of patients on this treatment modality. ‘Patients-like-me’ infographics were subsequently developed to visualize one-, three-, and five-year survival probabilities for each treatment modality in the PtDA (shown in Fig. 3). Hospitalisation rates were visualized as the average yearly rate of hospital admissions and the average length of each admission (in days). This allows patients to compare short- and long-term survival probabilities between their treatment options, and gives them insight on what they can expect regarding hospitalisation for each treatment modality.

### The ‘Kidney Failure Decision Aid’ prototype

**Fig. 4:**
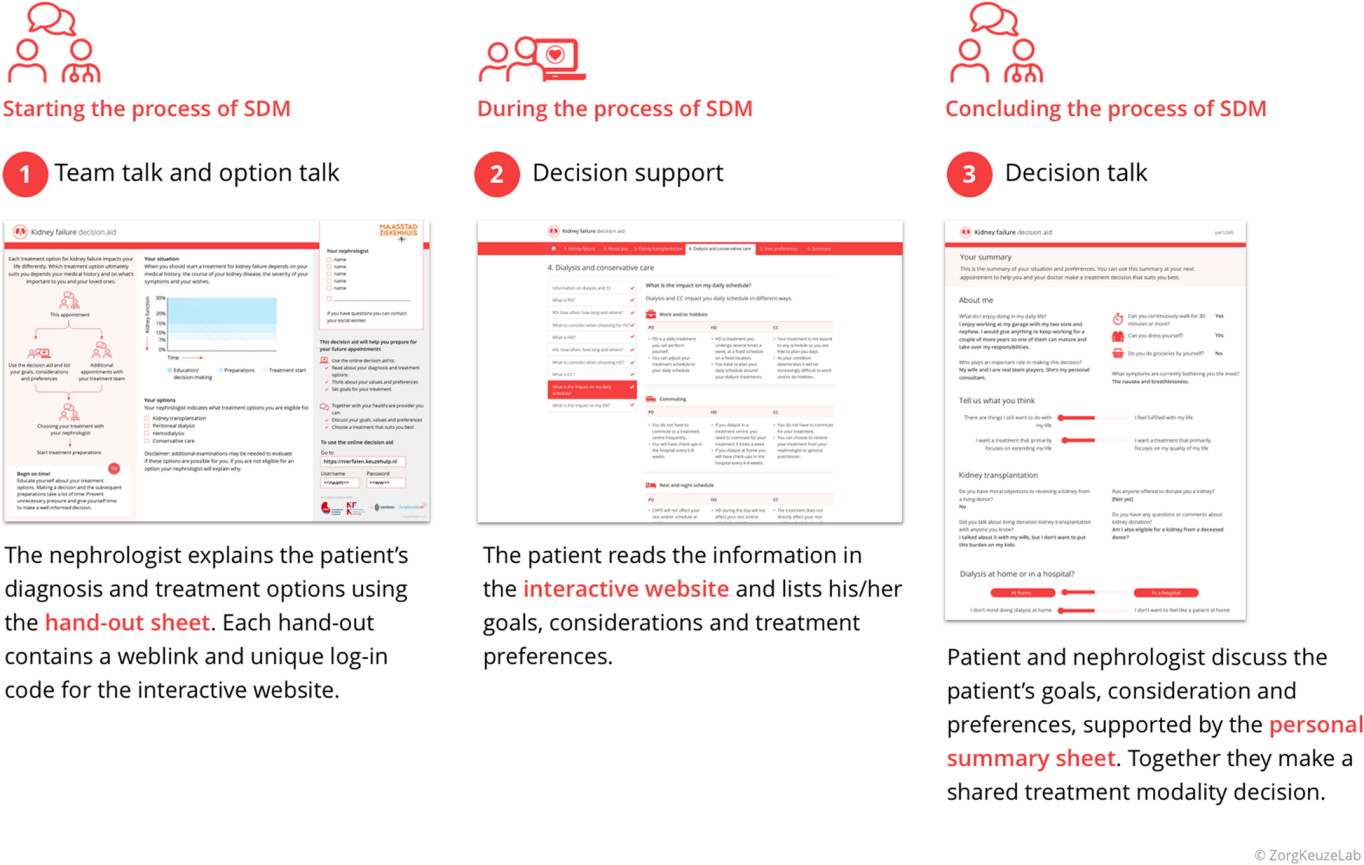
The three components of the ‘Kidney Failure Decision Aid’. *SDM* *= shared decision-making*. *Note: this is a translation from Dutch to English

The steering group developed a three-component PtDA, each of which was designed to support a different step of the SDM-process (shown in Fig. 4**)**:

1. The paper hand-out sheet (shown in Supplementary Figure S8A): provides patients with a schematic overview of the educational and decisional process, a graph on which the course of the patient’s kidney function can be drawn, and checkboxes that can be used to show patients for which treatment modalities they qualify. It also provides patients with a username and password to gain access to the second component of the PtDA.
2. The interactive website (shown in Supplementary Figure S8B and S8C): provides patients with educational content, real-world outcome information, patient reported outcome measures (PROMs) and values-clarification and preferences-elicitation exercises. This interactive website can be used in parallel with standard educational programmes in the Netherlands.
3. The personal summary sheet (shown in Supplementary Figure S8D): provides patients and clinicians with an overview of patients’ answers to the PROMs and exercises on the website. This summary sheet is automatically generated upon completion of the PtDA, after which patients can choose to share it with their clinicians.

The ‘Kidney Failure Decision Aid’ and all of its contents were written according to the B1 level of the Common European Framework of Reference for Languages (CEFRL) to ensure readability and comprehensibility among socioeconomically and educationally diverse patient populations. The interactive website was organized in chapters that follow a similar structure, discussing the basics first and providing more in-depth information as patients progress (shown in Supplementary Material S9). It also contains educational text and videos from nieren.nl, and videos from nierwijzer.nl that showcase both positive and negative patient experiences for each treatment modality. Moreover, each chapter contains links that redirect patients to different pages of these websites that provide additional information and videos on the respective subject.

### Alpha testing

Alpha testing of the prototype revealed that all of the identified needs and preferences among patients and clinicians (shown in Table 3), with the exception of two on education and decision-making, were met (shown in Table 4). Moreover, all treatment outcomes considered moderately useful, or very useful, were incorporated in the prototype (shown in Supplementary Material S10). Patient reported levels of fatigue and depression were not incorporated (shown in Supplementary Material S10). The ‘Kidney Failure Decision Aid’ prototype met all of the IPDAS minimum standards criteria and the IPDAS criteria for internet based PtDAs (shown in Supplementary Materials S11 and S12). All connections to the interactive website are secured by means of encryption and no identifying information is registered. Moreover, the hosting provider is ISO27001 and SOC type II certified.

**Table 3:** Needs and preferences of focus group participants on treatment modality education and decision-making, SDM, online PtDAs and outcome information

Topic	Needs and preferences	
	Patients and Caregiver	Clinicians
Education and decision-making	Don’t forget the person behind the patient* Clearly define the patient journey and take the lead as primary practitioner* Provide mentorship and guidance throughout the educational and decision-making process*	Coordinate with colleagues and adjust the education based on the educational needs of patients* Evaluate how well patients understand the provided information* Explore how patients make their choices, and who was involved in the decision-making process*
SDM	Strive for an equal patient-physician relationship* Facilitate patients in preference elicitation and values-clarification* Explicitly communicate when the decision has to be made*	Explicitly communicate that the opinions and wishes of patients are important in the decision* Do not try to “sell” any treatment modality, even if they have superior medical outcomes*
Online PtDA	Exercise caution for “informational overload”* Strive for collaboration and integrate everything on one platform*	Consider clinical practice when designing the PtDA* Pay attention to culture and health literacy*
Outcome information	Give patients autonomy in viewing outcome information* Provide tailored outcome information when possible*	Provide guidance on the interpretation of treatment outcomes* Pay attention to data visualization*

*SDM* = *Shared decision-making, PtDA* = *Patient decision aid.* *Note: this is a translation from Dutch to English

**Table 4:** Whether or not the ‘Kidney Failure Decision aid’ meets the needs and preferences identified in the focus group data

Topic	Needs and preferences identified in focus groups	Incorporated in the PtDA?
Education and decision-making	Don’t forget the person behind the patient*	Paper hand-out sheet, interactive website and summary sheet contain components that facilitate conversation on the personal situation, wishes, and preferences of patients
	Clearly define the patient journey and take the lead as primary practitioner*	Paper hand-out sheet contains a flowchart of the advanced CKD care pathway Interactive website contains a chapter with information on all involved clinicians, and when the decision has to be made
	Provide mentorship and guidance throughout the educational and decision-making process*	Paper hand-out sheet, interactive website, and summary sheet contain components that guide patients and clinicians in making values and preference-based decisions
	Coordinate with colleagues and adjust the education based on patients’ educational needs*	No
	Evaluate how well patients understand the provided information*	No
	Explore how patients make their choices, and who was involved in the decision-making process*	Interactive website and summary sheet contain components that facilitate conversations on who plays an important role in making decisions
SDM	Strive for an equal patient-physician relationship*	Paper hand-out sheet, interactive website, and summary sheet explicitly mention that the decision should be made according to the principles of SDM
	Facilitate patients in preference elicitation and values-clarification*	Paper hand-out sheet, interactive website and summary sheet contain components that facilitate conversation on the personal situation, wishes, and preferences of patients Paper hand-out sheet, interactive website, and summary sheet contain components that guide patients in values-clarification and preference-elicitation
	Explicitly communicate when the decision has to be made*	Paper hand-out sheet contains a flowchart of the advanced CKD care pathway Interactive website contains a chapter with information on all involved clinicians, and when the decision has to be made
	Explicitly communicate that the opinions and wishes of patients are important in the decision*	Paper hand-out sheet, interactive website, and summary sheet explicitly mention that the decision should be made according to the principles of SDM
	Do not try to “sell” any treatment modality, even if they have superior medical outcomes*	Paper hand-out sheet, interactive website, and summary sheet explicitly mention that the decision should be made according to the principles of SDM
Online PtDA	Exercise caution for “informational overload”*	Paper hand-out sheet contains elements that provide guidance on what chapters of the interactive website patients should focus on most Interactive website allows for easy navigation between chapters, saves patients’ location and answers when logging off, and provides the educational content in similarly structured chapters
	Strive for collaboration, and integrate everything on one platform*	PtDA has been developed in collaboration with key stakeholders and contains educational content of *nieren.nl* and *nierwijzer.nl*, both of which are owned by the Dutch kidney patient association and are endorsed by nephrology clinicians in the Netherlands
	Consider clinical practice when designing the PtDA*	PtDA has been developed for integration in established healthcare pathways without interfering with routine procedures
	Pay attention to health literacy and culture*	All components of the PtDA have been written in the B1 level of the CEFRL
Outcome information	Give patients autonomy in viewing outcome information*	Patients have to actively click through disclaimers before viewing survival probabilities and hospitalisation rates
	Provide tailored outcome information when possible*	Survival probabilities and hospitalisation rates have been stratified in age categories used by Dutch dialysis and kidney transplantation data registries
	Provide guidance on the interpretation of treatment outcomes*	Interactive website provides information on how patient characteristics impact treatment outcomes, and encourages conversations on treatment outcomes between patient and clinicians
	Pay attention to data visualization*	Outcome information has been visualised with infographics when possible

*PtDA* = *Patient decision aid, SDM* = *Shared decision-making, CEFRL* = *Common European Framework of Reference for Languages.* *Note: this is a translation from Dutch to English

### Beta testing

Seven patients and eight clinicians participated in the think-aloud sessions. None of these participants were involved in the needs-assessment or developmental process.

Patients had a mean age of 59 years and all of them (100%) were male (shown in Supplementary Material S13). Clinicians had a mean age of 39 years and three-quarters (75%) were female (shown in Supplementary Material S13).

Patients and clinicians noted that the prototype contained a lot of information, but patients indicated that it was all essential to make an informed choice. They also valued that it provided all the information on one platform and that educational content and videos from nieren.nl and nierwijzer.nl were embedded on the website. Some patients expressed difficulty in navigating the website due to navigational buttons blending in with background elements. Some patients were surprised by the confrontational nature of some questions (e.g. do you want your treatment to focus on extending your life or on the quality of your life?), but agreed that these were beneficial to the decision-making process. This was also the case for some treatment outcomes (e.g. survival probabilities). A few patients decided not to view these outcomes, but noted that every patient should have the right to choose whether they want to view this kind of information. Clinicians valued that the paper hand-out sheet could be used to express the urgency of the situation, and to show patients for which treatment options they qualify. Some clinicians expressed scepticism on whether their colleagues would use the graph on the paper hand-out sheet as intended. Some were afraid that patients could lose their username and password, or forget to bring their summary sheet to subsequent appointments. Both patients and clinicians believed that the personal summary sheet would support them in having meaningful conversations with one another, and help them make values- and preferences-based decisions. Illustrative quotations reflecting these findings have been extracted from the data (shown in Supplementary Material S14).

### Finalising the ‘Kidney Failure Decision Aid’

The steering group used the results from the beta testing to finalise the PtDA before implementation in clinical practice. Apart from some minor changes and additions, no major revisions were needed. Navigational buttons on the interactive website were made bigger and brighter to improve user-friendliness. In addition, a button that enables patients to directly send their personal summary sheet to their clinicians was integrated on the website.

### Implementation

The steering group developed a multi-faceted implementation strategy that consists of the following components:

- An introductory session for clinicians about SDM and using outcome information to support this process;
- Two e-learning courses about SDM and outcome information (“SDM with patients”, and “Applying outcome information in SDM”) for clinicians;
- In-person education and conversational skills training (“Conversational skills for SDM and the use of outcome information) for clinicians, during which they are thought and practice (with actors) conversational skills to successfully engage their patients in SDM and support this process with outcome information;
- Collaborating with clinicians to integrate the PtDA in local care pathways;
- Kick-off meetings to plan a formal ‘launch’ date and instruct clinicians on how to use the PtDA;
- Offering centralized implementation support and technical assistance for clinicians and patients;
- Assigning ‘local ambassadors’ to closely monitor progress and stimulate implementation through an implementation dashboard;
- A post-launch ‘refresher’ e-learning (“conversational skills for SDM and the use of outcome information”) for clinicians on the taught conversational skills.

Implementation of the ‘Kidney Failure Decision Aid’ has started in October 2020. The PtDA was implemented in a stepwise manner, starting with a lead and a co-lead hospital, to gain experience for subsequent implementation rounds. It has been handed out 304 times during its first year of use in the Santeon hospitals that have a pooled yearly incidence of approximately 350 patients with kidney failure.

## Discussion

The ‘Kidney failure Decision Aid’ was developed for patients that have to make kidney failure treatment modality decisions. It provides patients with real-world information on patient relevant outcomes, and consists of three components designed to facilitate SDM in clinical practice. We affirm the compatibility of the IPDAS development process model with a user-centred approach for the development of PtDAs [30]. The involvement of end-users and other key stakeholders was invaluable in: 1) gaining insight on their needs and preferences regarding the design and content of the PtDA, 2) establishing ongoing collaboration with the Dutch kidney patient association and national Dutch data registries that provided essential content for its development, and 3) developing an effective implementation strategy.

Most of the clinicians that participated in our needs-assessment indicated that they were satisfied with the education and decisional support they could provide patients in the advanced CKD care pathway. Likewise, most patients were satisfied with the education they had received in the past, and the majority agreed that it had sufficiently helped them in making treatment modality decisions. These findings are consistent with a previous survey study that showcased low rates of regret about the decision to start dialysis among patients in the Netherlands [31]. This could explain why, in our needs-assessment, the proportion of patients that considered a new online PtDA beneficial to the decision-making process was markedly smaller compared to the proportion of clinicians that considered it beneficial. However, we cannot disregard that this difference could also be explained by a response bias (e.g. desirability bias) among the participating clinicians. Shared decision-making and the use of PtDAs have received increasing national attention in the Netherlands over the past years, and clinicians might have felt obliged to provide desirable answers in the survey. Moreover, clinicians may already have been aware of the potential impact that PtDAs have on the decision-making process. Several publications have demonstrated that patients who use PtDAs (e.g. the PREPARED, iChoose kidney, or Yorkshire Dialysis Decision Aid) are generally more knowledgeable about their treatment options, and have better scores of decisional quality and patient activation compared to patients that do not use PtDAs [32–34].

The proportion of patients that wished to have PROMS in the PtDA was also smaller compared to clinicians. This was also the case for stories and experiences of peer-patients, and the impact of each treatment modality on social functioning and personal life. A recent systematic review that explored priorities of CKD patients relating to outcomes demonstrated that the most emphasised and feared outcome among patients were: 1) reaching kidney failure, and 2) having to choose between dialysis, transplantation and CCM [16]. This could explain why the patients that participated in our needs-assessment initially prioritized the basics, and more preferred to have the PtDA contain a detailed description of all treatment modalities alongside information regarding the pros and cons of each treatment modality, compared to PROMS, stories and experiences from peer-patients, or information about the impact of each treatment modality on social functioning and personal life.

Our collaboration with the Dutch kidney patient association and national Dutch outcome registries enabled us to develop a PtDA that contains all essential information for patients on one platform, and is easily used in parallel with standard educational programmes in the Netherlands. It also provided us with the survival data that we used to develop a ‘patients-like me’ infographic that we incorporated in the PtDA. In addition, our partnership with the Santeon hospitals enabled us to incorporate all other treatment outcomes that patients considered useful in the PtDA. These hospitals systematically measure and assess treatment outcomes in quality improvement cycles with the goal of improving patient care [25]. However, not all data was detailed enough to design ‘patients-like-me’ infographics for all treatment outcomes. We are now collaborating with other researchers currently collecting these data in the Netherlands, and plan on incorporating these in the PtDA as ‘patients-like-me’ infographics in the future. Another limitation is that patients can only use the PtDA if they are proficient in Dutch, and have the means and (digital) health-literacy necessary to navigate the interactive website. We actively encourage that clinicians instruct caregivers to help patients navigate the PtDA when they express difficulties in doing so. Moreover, the Dutch CKD care pathway also allows social workers and nurse practitioners to prompt patients to use the PtDA during intake appointments, and help patients use it when necessary. This creates an opportunity to help patients better prepare for: 1) future in-hospital educational appointments, and/or 2) consultations were the decision will be made. Similarly, online PtDAs can be used to help standardise treatment modality education when proprietary programs are inconsistent or lacking, allowing clinicians to work more efficiently and really engage their patients in deliberation and decision-making rather than having to educate their patients during consultations. Clinicians can also use online PtDAs to help educate patients that aren’t as mobile (e.g. due to physical disabilities) and/or live in rural areas [35]. Even though data have suggested that a large proportion of CKD patients have limited digital health literacy scores, many patients (especially minorities) are interested in using online health services [36]. Concurrently, these online health services (e.g. an online PtDA) have the potential to reduce disparities in healthcare, especially when they are part of multicomponent interventions that also include facility level policy and protocol changes [37].

We are among the first that asked patients to evaluate outcomes derived from the ICHOM and SONG standard sets on their usefulness for kidney failure treatment modality decision-making. The flexibility of each treatment modality, the survival of each treatment modality after treatment initiation, and the effect of each treatment modality on the residual kidney function over time were considered useful for this decision by the majority of patients. The least number of patients considered patient-reported levels of depression useful. This contrasts the ICHOM CKD standard set report [22] where patients considered depression a highly important outcome, and highlights the value of conducting a needs-assessment when it has yet to be determined what specific patients need for specific decisions. We had accordingly planned to perform focus groups with elderly and culturally diverse patients. We hypothesize that these patients have different needs and preferences regarding PtDAs and the use of outcome information for decision-making [38–40]. However, we were unable to perform these focus groups due to the first wave of the COVID-19 pandemic. We encourage other researchers to explore this hypothesis in future projects.

The ‘Kidney Failure Decision Aid’ does not suggest patients choose a particular treatment modality but rather encourages patients and clinicians to engage in conversation and make decisions based on patients’ values and preferences. The steering group wanted the PtDA to focus on helping patients make decisions through the principles of SDM and our beta testing indicated that patients and clinicians believed that it would help patients make informed decisions, and that it would support values- and preferences-based decision-making. We are currently investigating the effectiveness of the ‘Kidney Failure Decision Aid’ in an interrupted time-series design study (Netherlands Trial Register no. NL8376) [41]. This study will provide preliminary evidence of its effects on SDM and other decision-making outcomes as perceived by patients.

## Conclusions

In conclusion, we developed an online PtDA to support kidney failure treatment modality decisions that provides patients with real-world outcome information and facilitates SDM in clinical practice. The IPDAS development process model for PtDAs is compatible with user-centred design methods. The effects of the ‘Kidney Failure Decision Aid’ on SDM and other decision-making outcomes are currently being investigated in a clinical trial. Future studies should investigate the usefulness of real-world outcome information for kidney failure treatment modality decision-making in elderly and culturally diverse patient populations.

## Supplementary Information

**Additional file 1.** Supplementary materials

## Data Availability

The datasets used and/or analysed during the current study are available from the corresponding author on reasonable request.

## Abbreviations

CKD: Chronic Kidney disease
SDM: Shared Decision-Making
DSI: Decision Support Intervention
PtDA: Patient Decision Aid
IPDAS: International Patient Decision Aids Standards
OHRI: Ottawa Hospital Research Institute
ICHOM: International Consortium for Health Outcome Measurement
SONG: Standardised Outcomes in Nephrology
NVN: Dutch kidney patient association
COREQ: Consolidated Criteria for Reporting Qualitative Research
KRT: Kidney Replacement Therapy
eGFR: Estimated Glomerular Filtration Rate
LDKT: Living Donation Kidney Transplantation
DDKT: Deceased Donation Kidney Transplantation
PD: Peritoneal Dialysis
HD: Haemodialysis
CCM: Conservative care management
PROMS: Patient Reported Outcome Measures
CEFRL: Common European Framework of Reference for Languages
