# Supplementary material for: Development of an online patient decision aid for kidney failure treatment modality decisions
Source: BMC Nephrol. 2022 Jul 6;23:236. doi: 10.1186/s12882-022-02853-0 (PMC9257566; doi:10.1186/s12882-022-02853-0)
Supplement: Supplementary file 1 — Additional file 1. Supplementary materials [file 12882_2022_2853_MOESM1_ESM.docx]

**Supplementary Material for: Development of the ‘Kidney Failure Decision Aid’**

**Supplementary Material S1**

**
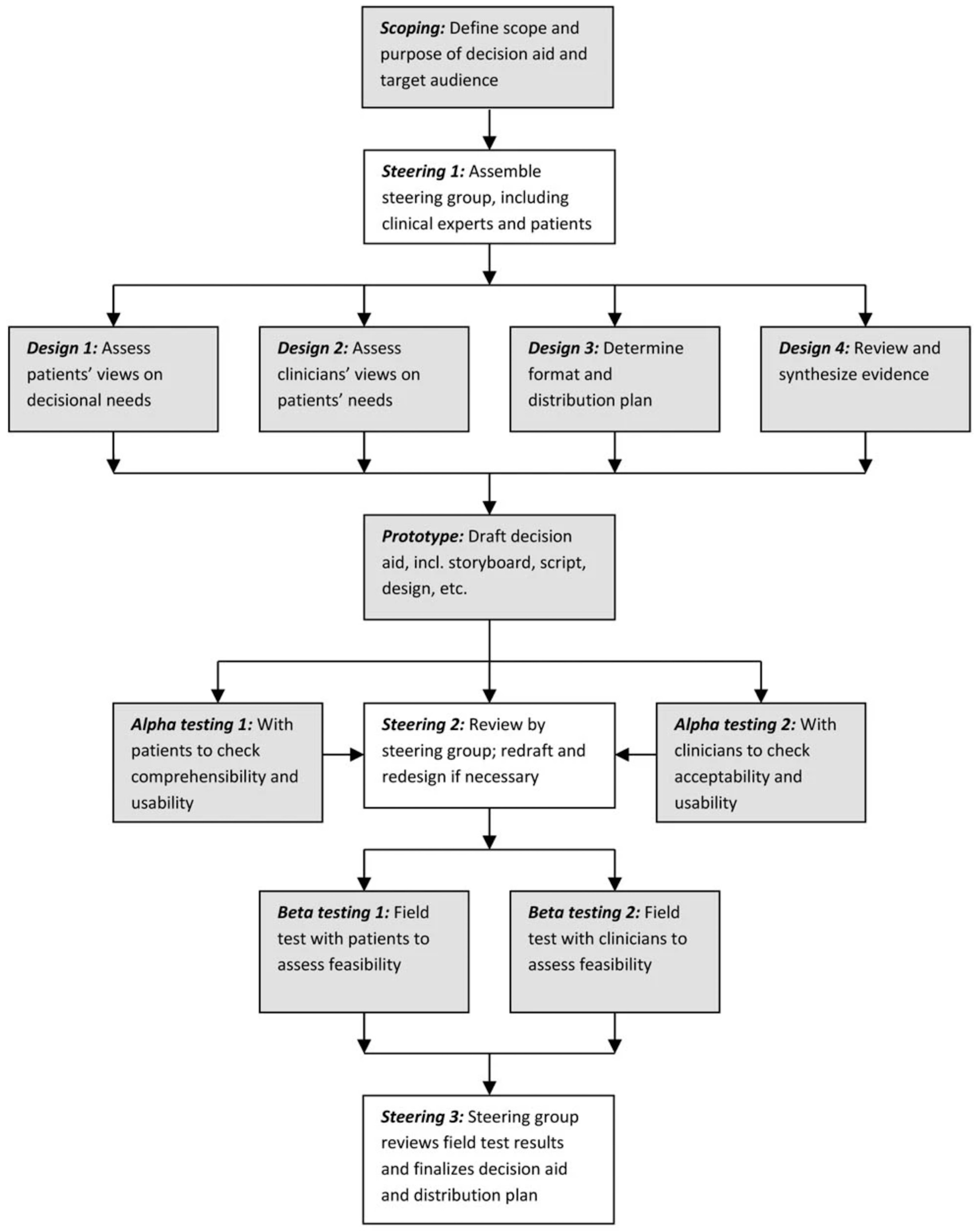
**Schematic overview of IPDAS developmental process (Coulter, et al. 2013)

**Supplementary Material S2**

Additional information on the online survey for patients:

The online survey contained a total of 34 self-constructed questions. The survey was tested and amended for face-validity by anonymous patients that were recruited by the Dutch kidney patient association. At the start of the survey, participants were presented with an explanatory text introducing the concept of SDM, outcome measures and an explanation of the intended purposes of the survey. After this, the survey contained questions on the basic demographics of the participants, and questions regarding their experiences with the provision of information for decision-making. After this, the survey contained questions regarding the perceptions and wishes of patients regarding an online PtDA. Finally, participants were presented with a list of 15 outcome measures that were formulated to encompass the essential and core outcomes for CKD, hemodialysis, peritoneal dialysis and kidney transplantation as formulated by ICHOM and SONG. Afterwards, patients were asked to indicate whether they considered these outcomes useful for treatment modality decision-making. Outcomes were ranked from most to least useful based on the proportion of patients that considered each outcome useful. We did not include quality of life as an individual outcome because patients indicated that it was too abstract when the surveys were tested for face-validity. Instead, we decided to include patient reported outcomes that capture the different domains of quality of life. The survey ended with statements regarding the consent of participants to be approached for participation in the focus groups.

Additional information on the online survey for clinicians:

The online survey for clinicians contained a total of 30 self-constructed questions. The survey was tested and amended for face-validity by involved experts on medical decision-making and the communication sciences (AS, NU). At the start of the survey, participants were presented with an explanatory text introducing the concept of SDM, outcome measures and a brief explanation of the intended purposes of the survey. After this, the survey contained questions on the basic demographics of the participants, and questions regarding their experiences with the provision of information for decision-making. After this, the survey contained questions on the perceptions and wishes of clinicians regarding an online PtDA. The survey ended with statements regarding the consent of participants to be approached for participation in the focus groups.

Additional information on the focus groups:

All focus groups were held according to a predetermined format. Each focus group was estimated to last approximately two hours. All focus group were guided by one moderator (NE) and two facilitators (EP, WK or RT). First, an overview on the topics and definitions were given after a few welcoming words. After this, a statement about the ground rules of the focus group was made. Participants were notified of the fact that everything was going to be audiotaped, and were given the assurance of confidentiality. Following on from this, introductory discussions were assisted by persona descriptions. Afterwards, in-depth discussions were held on the experiences and preferences of participants with treatment modality education and decision-making, PtDAs, SDM, and how outcome information can be used to support decision-making. Finally, all participants were asked to fill out questionnaires regarding their basic demographics.

**Supplementary Material S3**

Information on the demographic characteristics of patients and clinicians that participated in the online surveys

| **Demographic characteristics** | **Patients** | **Clinicians** |
| --- | --- | --- |
| Age (in years) | Mean = 59 (SD = 12) | Mean = 48 (SD = 11) |
| Sex | Male = 66 (53%)  Female = 59 (47%) | Male = 12 (29%)  Female = 29 (69%)  Gender neutral = 1 (2%) |
| Immigration status | Native Dutch = 117 (94%)  Non-native Dutch = 8 (6%) | Native Dutch = 36 (86%)  Non-native Dutch = 6 (14%) |
| Civil status | Not alone = 97 (78%)  Alone = 28 (22%) | N/A |
| Educational level | Low = 24 (19%)  Middle = 35 (28%)  High = 66 (53%) | N/A |
| Active treatment modality | In-centre HD (day) = 15 (12%)  In-centre HD (night) = 5 (4%)  Home HD = 4 (3%)  CAPD = 1 (1%)  APD = 4 (3%)  LDKT = 54 (43%)  DDKT = 32 (26%)  CCM = 3 (2%)  No active treatment (yet) = 7 (6%) | N/A |
| Profession | N/A | Nephrologist = 15 (36%)  NP / PA = 8 (19%)  RN = 9 (21%)  Social worker = 10 (24%) |
| Working experience (in years) | N/A | Mean = 11 (SD = 9) |
| Exposure to patients with ESKD (monthly amount) | N/A | 0 = 1 (3%)  1 – 5 = 17 (48%)  5 – 10 = 3 (9%)  > 10 = 14 (40%) |
| Duration of treatment modality education and decision making (in months) | N/A | Mean = 4 (SD = 3) |

N/A = not applicable. HD = haemodialysis, CAPD = continuous ambulatory peritoneal dialysis. APD = ambulatory peritoneal dialysis. LDKT = living donation kidney transplantation. DDKT = deceased donor kidney transplantation. CCM = conservative care management. NP = nurse practitioner. PA = physician assistant. RN = registered nurse. ESKD = end-stage kidney disease.

**Supplementary Material S4**

Information on how participating clinicians educate their patients on their treatment options

| **How clinicians educate their patients** | **Result / outcome** |
| --- | --- |
| During outpatient consultations with a nephrologist. | Yes = 32 (97%)  No = 1 (3%) |
| During outpatient consultations with a NP / PA. | Yes = 17 (52%)  No = 16 (48%) |
| During outpatient consultations with a RN. | Yes = 30 (91%)  No = 3 (9%) |
| During outpatient consultations with a social worker. | Yes = 30 (91%)  No = 3 (9%) |
| By referring patients to a special educational centre. | Yes = 4 (12%)  No = 29 (88%) |
| By handing out proprietary educational printed materials. | Yes = 16 (49%)  No = 17 (51%) |
| By handing out third-party educational printed materials. | Yes = 23 (70%)  No = 10 (30%) |
| By referring patients to a proprietary educational website. | Yes = 8 (24%)  No = 25 (76%) |
| By referring patients to NVN.nl (official website of the Dutch kidney patient association). | Yes = 10 (30%)  No = 23 (70%) |
| By referring patients to nierstiching.nl (official website of the Dutch kidney foundation). | Yes = 16 (52%)  No = 17 (48%) |
| By referring patients to nierwijzer.nl (website that contains a video library of interviews with more than 40 patients with kidney disease. Owned by the Dutch kidney patient association). | Yes = 21 (64%)  No = 12 (36%) |
| By referring patients to nieren.nl (educational website for patients with kidney disease. Owned by the Dutch kidney patient association). | Yes = 19 (58%)  No = 14 (42%) |
| By referring patients to other third-party educational websites. | Yes = 2 (6%)  No = 31 (94%) |
| By handing out a PtDA. | Yes =1 (3%)  No = 32 (97%) |
| Are you satisfied with the educational services provided in your centre? | Very unsatisfied = 0 (0%)  Unsatisfied = 4 (12%)  Neutral = 5 (15%)  Satisfied = 23 (70%)  Very satisfied 1 (3%) |
| Our patients get enough information to make an informed decision. | Completely disagree = 0 (0%)  Disagree = 2 (6%)  Neutral = 1 (3%)  Agree = 22 (69%)  Completely agree = 7 (22%) |
| I feel the information on treatment options that is accessible to patients is reliable. | Completely disagree = 0 (0%)  Disagree = 1 (3%)  Neutral = 1 (3%)  Agree = 24 (75%)  Completely agree = 6 (19%) |

NP = nurse practitioner. PA = physician assistant. RN = registered nurse. PtDA = patient decision aid*.* ***Note: this is a translation from Dutch to English.**

**Supplementary Material S5**

Information on how patients felt about their treatment options education

| **Statements** | **Result / outcome** |
| --- | --- |
| Reliable information on the different treatment modalities was easy to find. | Completely disagree = 8 (6%)  Disagree = 11 (9%)  Neutral = 26 (21%)  Agree = 54 (43%)  Completely agree = 25 (21%) |
| The information supported me enough to make a treatment modality decision. | Completely disagree = 9 (7%)  Disagree = 9 (7%)  Neutral = 16 (13%)  Agree = 65 (52%)  Completely agree = 26 (21%) |
| I feel I had enough knowledge regarding my options when I had to choose my treatment modality. | No = 24 (20%)  Yes = 97 (78%)  I don’t know = 3 (2%) |

***Note: this is a translation from Dutch to English.**

**Supplementary Material S6**

Information on the demographic characteristics of patients, the caregiver and clinicians that participated in the focus groups

| **Demographic characteristics** | **Patients / Caregiver** | **Clinicians** |
| --- | --- | --- |
| Age (in years) | Mean = 51 (SD = 17) | Mean = 52 (SD = 8) |
| Sex | Male = 5 (63%)  Female = 3 (37%) | Male = 2 (22%)  Female = 7 (78%) |
| Immigration status | Native Dutch = 7 (88%)  Non-native Dutch = 1 (12%) | Native Dutch = 5 (56%)  Non-native Dutch = 4 (44%) |
| Civil status | Not alone = 6 (75%)  Alone = 2 (25%) | N/A |
| Educational level | Low = 1 (12%)  Middle = 1 (12%)  High = 6 (76%) | N/A |
| Active treatment modality | In-centre HD (day) = 1 (14%)  LDKT = 6 (86%) | N/A |
| Profession | N/A | Nephrologist = 2 (22%)  NP / PA = 2 (22%)  RN = 2 (22%)  Social worker = 3 (34%) |
| Working experience (in years) | N/A | Mean = 7 (SD = 7) |

N/A = not applicable. HD = haemodialysis. LDKT = living donation kidney transplantation. NP = nurse practitioner. PA = physician assistant. RN = registered nurse*.*

**Supplementary Material S7**

Quotations of patients, the caregiver and clinicians supporting the themes listed in Table 3

| **Education and decision-making** | **Quotation(s)** |
| --- | --- |
| Don’t forget the person behind the patient. | “Everyone said that I should do CAPD instead of HD and I hated that. Finally, instead of my own nephrologist, another doctor came to talk to me. I noticed immediately that his goal was to change my mind. He had a counter-argument for every argument I had, until I finally said “but I really like going to the beach”, and he said “but they have very nice bathing suits nowadays”, and I said “but I always go to the nude beach”. He didn’t have an answer to that anymore, and said “we’re going to schedule a fistula operation”. (Patient). |
| Clearly define the patient journey and take the lead as primary practitioner. | “We really felt like we were left to our fate. The nephrologist was often unaware of the situation with the urologist and vice-versa. It seemed like they were working past each other. We really had to do our best and convince the nephrologist to contact and pressure the urologist to plan the operation”. (Caregiver) |
| Provide mentorship and guidance throughout the educational and decision-making process. | “I always take my partner with me to my appointments because I know that I am not very assertive. I think that it is important to have someone help you prepare for the conversations you are about to have. Maybe even someone who can be present during these conversations. Perhaps it would be an idea to have someone from the hospital, like a social worker, accompany you throughout the process”. (Patient). |
| Coordinate with colleagues and adjust the education based on patients’ educational needs. | “I think that it is a good thing that we give patients information on all the treatment modalities. Nevertheless, I think we could improve this educational process by consulting with each other more often: who does what, when and how? Do we continue? Or is there still a need for something more?”. (Clinician). |
| Evaluate how well patients understand the information provided. | “I think it would be a good idea to evaluate how much patients remember of the conversations they’ve had when you see them again. I often notice that they’ve forgotten a lot when they come back to me. I think something like a time-out appointment would be beneficial to us all”. (Clinician). |
| Explore how patients make their choices, and who was involved in the decision-making process. | “I think it’s really important to know how they came to their decision, regardless of their choice. Has someone made the decision independently, or were other people involved? I recently had a young man who was thinking about starting with PD, as he is still very active and works regular hours. He said he was going to think about it, and talk about it with his daughter. After some time he came back and said he had chosen for HD. Apparently he really values the opinion of his daughter, and I would’ve liked to know that beforehand because then I would’ve asked her to join him during his educational sessions”. (Clinician). |
| **SDM** | **Quotation(s)** |
| Strive for an equal patient-physician relationship. | “You have to be able to talk openly with each other, on the same level. It’s not like, he knows because he’s the doctor so I just have to accept it. No, you have to able to really communicate with each other.” (Patient). |
| Facilitate patients in preference elicitation and values-clarification. | “I think that it’s a two-way street between patient and clinician. I know people that go to the doctor and are satisfied with hearing: ‘take this pill and we’ll see each other in 3 months”. That’s ok, but I think you will never build a meaningful relationship with your doctor that way. How will you ever talk about your hobbies or what matters to you like this?” (Patient). |
| Explicitly communicate when the decision has to be made. | “Of course it’s difficult because you have people who are on pre-dialysis for ten years, and you have people whose kidneys deteriorate quickly and need treatment within three months. At some point you have to decide. After that it’s a waiting game, but at least you’ll know for what.” (Patient). |
| Explicitly communicate that the opinions and wishes of patients are important in the decision. | “I always tell patients: “You’ll get education on your treatment options and after that we’ll make decision. But first let’s talk about your options, what you think about them and what really fits best in your life, and. After that, we’ll make a decision together.” (Clinician) |
| Do not try to “sell” any treatment modality, even if they have superior medical outcomes. | “We always try to emphasize the positive aspects of the different treatment modalities during their education. But I think it’s important to try and not persuade people. Imagine you convince someone to choose for something and it doesn’t go as planned. I would feel very guilty. It really has to be their own decision.” (Clinician) |
| **Online PtDA** | **Quotation(s)** |
| Exercise caution for “informational overload”. | “I just don’t want to get too much information at once. When you become a patient you already have to process so much. I don’t want to get bombarded with information. I would just not understand anything anymore, get frustrated and then nothing would work for me.” (Patient). |
| Strive for collaboration, and integrate everything on one platform. | “It would be great if there was one address that contained all the information we need to make a decision. Now we know where all the relevant information can be found, but if you’re just starting as a patient you have no clue at all. With a tool like this, you just get if from your doctor and log in. I think that’s great.” (Patient). |
| Consider clinical practice when designed the PtDA. | “And if someone completes the PtDA, will there be link to their answers or something? There should be a way for clinicians to see patient’s answers without losing too much time.” (Clinician). |
| Pay attention to health literacy and culture. | “I really think you should keep it as simple as possible. Remember that there’s quite some patients that aren’t very literate. It would be difficult for them to understand lots of text. Integrating images or videos in the PtDA could really help in my opinion.” (Clinician). |
| **Outcome information** | **Quotation(s)** |
| Provide tailored outcome information when possible. | “To answer the question on whether or not I would like to see this information, my answer would be yes. But then I want customization. I want to log in, and have all the data relevant to my situation without having to click too much.” (Patient). |
| Give patients autonomy in viewing outcome information. | “If I have to choose something I should be able to weigh and compare the risks of my options. But I think that if you just become a kidney patient, you’ll be scared to death when confronted with information on certain outcomes You don’t have to see everything at once, but it should be a possibility when you’re ready for it. I mean, you can find almost everything on the internet anyway.” (Patient). |
| Pay attention to data visualization. | “I think that a visual representation of a risk instead of a mean or percentage would be more insightful to patients. Like three of ten figures that become red, instead of writing 30%.” (Clinician). |
| Provide guidance on the interpretation of treatment outcomes. | “You know, I think that if people want to know something, they’re going to find it anyway. I think that it’s a positive thing to integrate outcome information in a PtDA. But I think that apart from providing patients with this information, our role should be to help patients understand it.” (Clinician). |

CAPD = continuous ambulatory peritoneal dialysis. PD = peritoneal dialysis. HD = haemodialysis. PtDA = patient decision aid. ***Note: this is a translation from Dutch to English***.*

**Supplementary Material S8A**

**
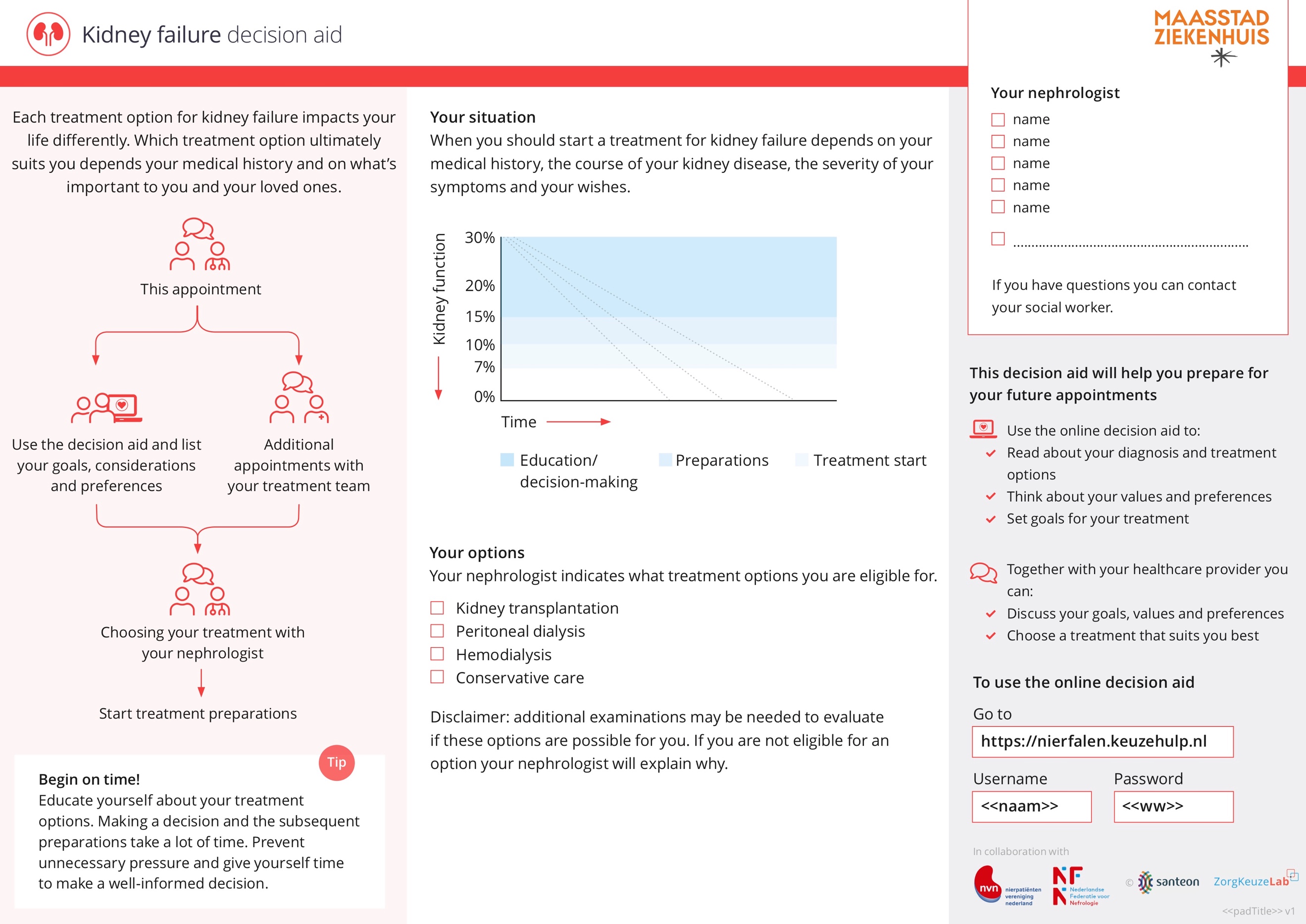
**

Paper hand-out sheet. ***Note: this is a translation from Dutch to English.**

**Supplementary Material S8B**

**
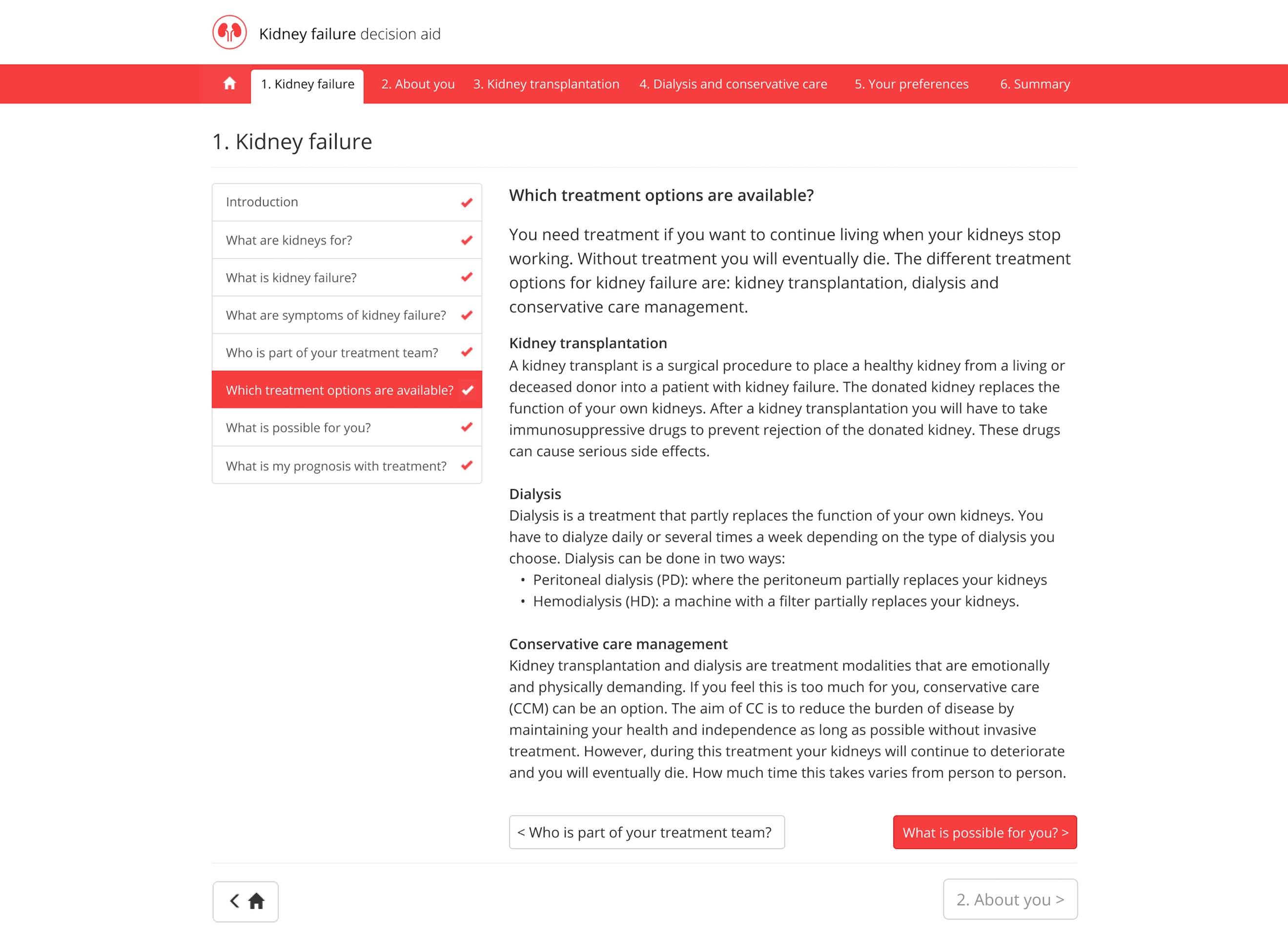
**

Webpage with educational content from interactive website. PD = Peritoneal Dialysis. HD = Haemodialysis. CCM = Conservative Care Management. ***Note: this is a translation from Dutch to English.**

**Supplementary Material S8C**

**
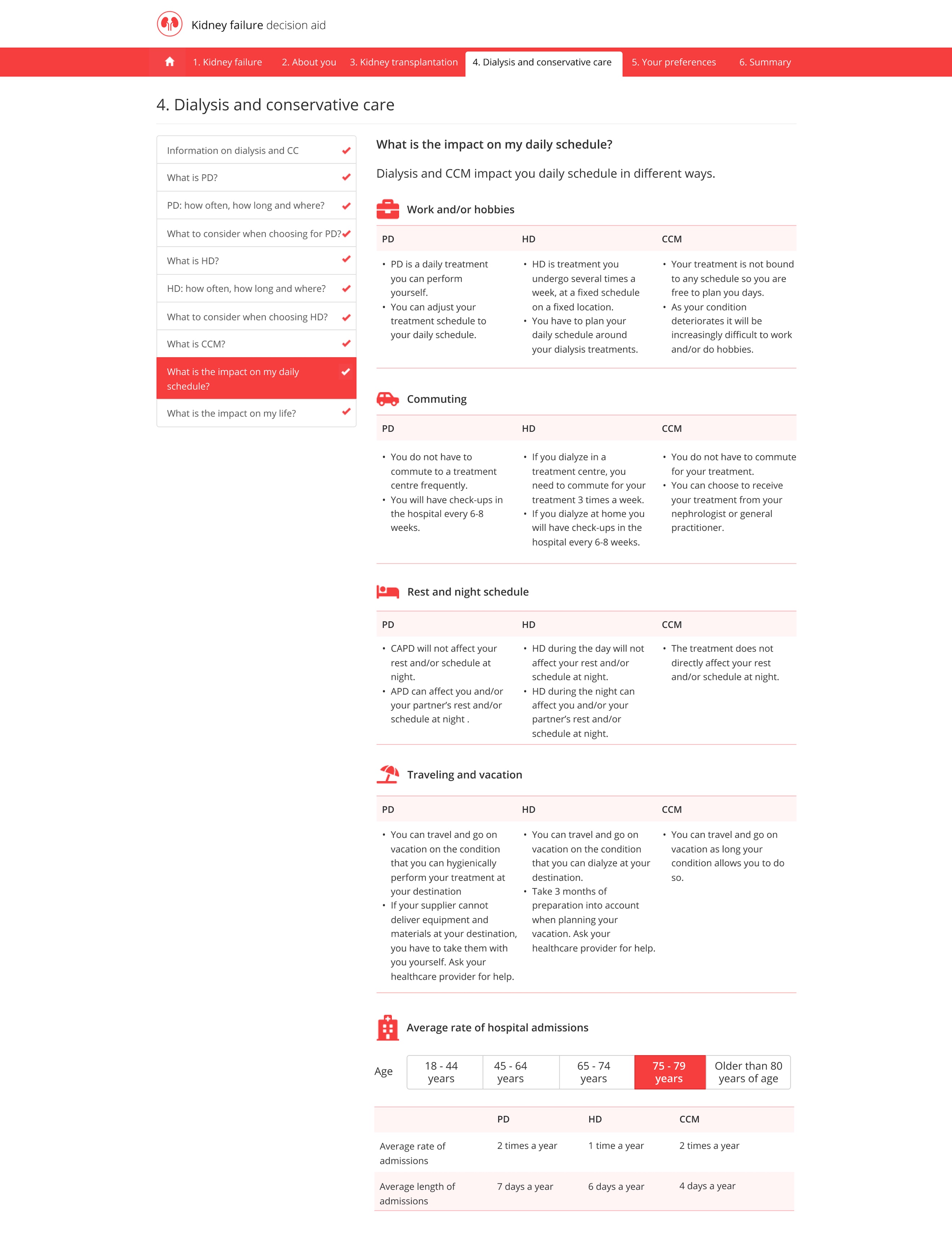
**

Webpage with educational content and real-world outcome information on hospitalisation rates per treatment modality. PD = Peritoneal Dialysis. HD = Haemodialysis. CCM = Conservative Care Management. ***Note: this is a translation from Dutch to English.**

**Supplementary Material S8D**

**
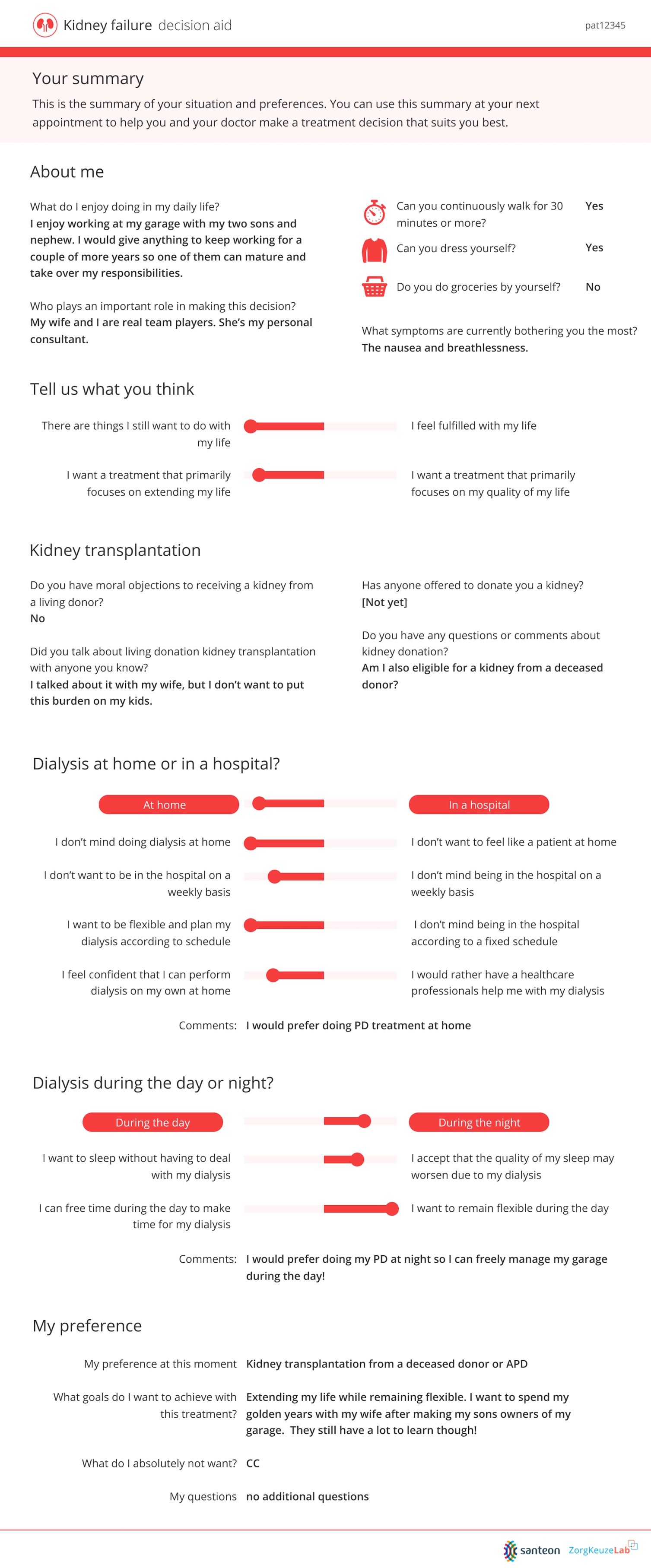
**

Personal summary sheet. ***Note: this is a translation from Dutch to English.**

**Supplementary Table S9**

Overview of the educational contents and questions in the ‘Kidney Failure Decision Aid’

| **Chapter** | **Topics discussed or questions asked** |
| --- | --- |
| 1. Kidney failure | 1. The basic anatomy and physiology of the kidneys. 2. The underlying mechanisms and classification of chronic kidney disease. 3. Kidney failure and its associated symptoms. 4. An overview of clinicians involved in the treatment of kidney failure. 5. A basic overview of all treatment options for kidney failure. 6. An explanation of factors that dictate eligibility for the respective treatment options. 7. Outcome information: survival per treatment modality. |
| 2. About you | 1. Open question: what do I enjoy doing in my daily life? 2. Open question: who plays an important role in making this decision? 3. Open question: can you continuously walk for 30 minutes or more? 4. Open question: can you dress yourself? 5. Open question: do you do groceries by yourself? 6. Open question: what symptoms are currently bothering you the most? 7. Likert scale: I feel fulfilled with my life vs. there are things I still want to do with my life. 8. Likert scale: I want a treatment that primarily focuses on my quality of life vs. I want a treatment that primarily focuses on extending my life. |
| 3.A. Kidney transplantation | 1. Background information on transplantation. 2. Why choose for transplantation? 3. LDKT vs. DDKT. 4. Additional information on factors that dictate eligibility for transplantation. 5. Factors that dictate the risk of rejection. 6. What happens after the operation. 7. The potential impacts of LDKT on the donor. |
| 3.B. Kidney transplantation: your considerations | 1. Open question: do you have moral objections to receiving a kidney from a living donor? 2. Open question: did you talk about living donation kidney transplantation with anyone you know? 3. Open question: has anyone offered to donate you a kidney? 4. Open question: do you have any questions or comments about kidney donation? |
| 4. Dialysis and CCM | 1. Background information on dialysis and CCM. 2. What is PD? 3. How often, when and where PD? 4. Why choose for PD? 5. What is HD? 6. How often, when and where HD? 7. Why choose for HD? 8. What is CCM? 9. Why choose for CCM? 10. How do these treatment modalities affect my daily schedule? 11. Outcome information: hospitalisation rates per treatment modality. 12. How do these treatment modalities affect my life? |
| 4.B. Dialysis at home or in a hospital? | 1. Likert scale: I don’t mind doing dialysis at home vs. I don’t want to feel like a patient at home. 2. Likert scale: I don’t want to be in the hospital on a weekly basis vs. I don’t mind being in the hospital on a weekly basis. 3. Likert scale: I want to be flexible and plan my dialysis according to my schedule vs. I don’t mind being in the hospital according to a fixed schedule. 4. Likert scale: I feel confident that I can dialysis on my own at home vs. I would rather have a healthcare professional help me with my dialysis. 5. Likert scale: I prefer dialysis at home vs. I prefer in-centre dialysis. 6. Open question: why do you prefer this dialysis modality? |
| 4.C. Dialysis during the day or night? | 1. Likert scale: I want to sleep without having to deal with my dialysis vs. I accept that the quality of my sleep quality may worsen due to my dialysis. 2. Likert scale: I can free time during the day to make time for my dialysis vs. I want to remain flexible during the day. 3. Likert scale: I prefer dialysis during the day vs. I prefer dialysis during the night. 4. Open question: why do you prefer this dialysis time? |
| 5. Your preferences | 1. Open question: my preference at this moment. 2. Open question: what goals do I want to achieve with this treatment? 3. Open question: what do I absolutely not want? 4. Open question: do you have any remaining questions you would like to discuss with your clinician? |
| 6. Summary | 1. Personal summary sheet is generated. |

HD = haemodialysis. LDKT = living donation kidney transplantation. DDKT = deceased donor kidney transplantation. CCM = conservative care management. ***Note: this is a translation from Dutch to English.**

**Supplementary Material S10**

Overview of which treatment outcomes were incorporated in the ‘Kidney Failure Decision Aid, and how they are presented

| **Treatment outcomes**  **1 = highest, 15 = lowest** | **Incorporated as** |
| --- | --- |
| 1. The flexibility of each treatment modality | Explanatory text for transplantation, PD/HD and CCM in the third and fourth chapters of the interactive website. |
| 1. The survival of each treatment modality after initiation | Explanatory text for transplantation and PD/HD in the third and fourth chapters of the interactive website. |
| 1. The effect of each treatment modality on the residual kidney function over time | Explanatory text for PD and HD in the fourth chapter of the interactive website. |
| 1. Patient reported levels of physical functioning on each treatment modality | Explanatory text and summarised information in tables for transplantation, PD/HD and CCM in the third and fourth chapters of the interactive website. |
| 1. The effects of each treatment modality on social functioning | Explanatory text and summarised information in tables for transplantation, PD/HD and CCM in the third and fourth chapters of the interactive website. |
| 1. Patient survival on each treatment modality | ‘Patients-like-me’ infographics in the first chapter of the interactive website. |
| 1. The effects of each treatment modality on personal life | Explanatory text and summarised information in tables for transplantation, PD/HD and CCM in the third and fourth chapters of the interactive website. |
| 1. Complication rates related to immunosuppressive drugs after transplantation | Explanatory text in the third chapter of the interactive website. |
| 1. Hospitalisation rates for each treatment modality | Event rates in the fourth chapter of the interactive website. |
| 1. Event rates for cardiovascular complications on each treatment modality | Explanatory text for PD and HD in the fourth chapter of the interactive website. |
| 1. Patient reported levels of pain on each treatment modality | Explanatory text for HD in the fourth chapter of the interactive website. |
| 1. Vascular access survival in HD | Explanatory text for HD in the fourth chapter of the interactive website. |
| 1. Patient reported levels of fatigue on each treatment modality | Not incorporated |
| 1. PD peritonitis rates | Event rates in the fourth chapter of the interactive website. |
| 1. Patient reported levels of depression on each treatment modality | Not incorporated |

HD = haemodialysis. PD = peritoneal dialysis. CCM = conservative care management. ***Note: this is a translation from Dutch to English.**

**Supplementary Material S11**

Score of the ‘Kidney Failure Decision Aid’ on the IPDAS minimum criteria standards set

|  | **IPDAS minimum standards criteria** | **Meets criterion?** |
| --- | --- | --- |
| **Qualifying criteria** | 1. The PtDA describes the health condition or problem for which for which the index decision is required. | Yes |
|  | 1. The PtDA explicitly states the index decision that needs to be considered. | Yes |
|  | 1. The PtDA describes the options available for the index decision. | Yes |
|  | 1. The PtDA describes the positive features of each option. | Yes |
|  | 1. The PtDA describes the negative features of each option. | Yes |
|  | 1. The PtDA describes what it is like to experience the consequences of the options. | Yes |
| **Certification criteria** | 1. The PtDA shows the negative and positive features of each option with equal detail. | Yes |
|  | 1. The PtDA (or associated documentation) provides citations to the evidence selected. | Yes |
|  | 1. The PtDA (or associated documentation) provides a production or publication date. | Yes |
|  | 1. The PtDA (or associated documentation) provides information about the update policy. | Yes |
|  | 1. The PtDA (or associated documentation) provides information about the levels of uncertainty around event or outcome probabilities. | Yes |
|  | 1. The PtDA (or associated documentation) provides information about the funding source used for development. | Yes |

PtDA = patient decision aid.

**Supplementary Material S12**

Score the on additional IPDAS criteria for PtDAs that are internet based

| **IPDAS criteria for internet-based PtDAs** | **Meets criterion?** |
| --- | --- |
| 1. The PtDA provides a step-by-step way to move through the web pages. | Yes |
| 1. The PtDA allows patients to search for key words. | Yes |
| 1. The PtDA provides feedback on personal health information that is entered into the PtDA. | Yes |
| 1. The PtDA provides security for personal health information entered into the PtDA. | Yes |
| 1. The PtDA makes it easy for patient to return to the decision aid after linking to other web pages. | Yes |
| 1. The PtDA permits printing as a single document. | Yes |

PtDA = patient decision aid.

**Supplementary Material S13**

Information on the demographic characteristics of patients and clinicians that participated in the beta testing

| **Demographic characteristics** | **Patients** | **Clinicians** |
| --- | --- | --- |
| Age (in years) | Mean = 59 (SD = 13) | Mean = 39 (SD = 6) |
| Sex | Male = 7 (100%) | Male = 2 (25%)  Female = 6 (75%) |
| Immigration status | Native Dutch = 7 (100%) | Native Dutch = 7 (88%)  Non-native Dutch = 1 (12%) |
| Civil status | Not alone = 5 (71%)  Alone = 2 (29%) | N/A |
| Educational level | Low = 1 (14%)  Middle = 3 (43%)  High = 3 (43%) | N/A |
| Active treatment modality | In-centre HD (day) = 1 (14%)  Home HD = 1 (14%)  APD = 2 (29%)  LDKT = 3 (43%) | N/A |
| Profession | N/A | Nephrologist = 6 (76%)  NP / PA = 1 (12%)  RN = 1 (12%) |
| Working experience (in years) | N/A | Mean = 6 (SD = 5) |

N/A = not applicable. HD = haemodialysis. LDKT = living donation kidney transplantation. APD = automated peritoneal dialysis. NP = nurse practitioner. PA = physician assistant. RN = registered nurse.

**Supplementary Material S14**

Quotations of patients and clinicians on the ‘Kidney Failure Decision Aid” and its respective components.

| **Component** | **Quotation(s) patients** | **Quotation(s) clinicians** |
| --- | --- | --- |
| Overall impressions | “The decision aid is well structured. I wish it had already been available when I was faced with this decision.”  “I see it as a manual that you can use to educate yourself, and come up with questions to discuss with your nephrologist. I also across certain topics that I didn’t even know were relevant to think about.”  “I would recommend other patients to use this decision aid because it contains everything you need to know in one place. The information on the different treatment modalities is clear, and the overview at the end helps in comparing them.” | “I think it is well structured and that it looks very nice. I am excited. I hope it will be used a lot.”  “The conversations I have with my patients are most important to me. I think this decision aid can help me with that.”  “The decision aid is a nice complement to our conversations. It really allows patients to take a step back and read up on what we have discussed.”  “How do we prevent people from losing their username and password, or coming to the outpatient clinic without their handout-sheet? |
| Paper hand-out sheet | “The graph on the paper hand-out sheet appeals to me. As simple as it is, with this drawing I can see my upper limit, my lower limit, and when something really has to be done.”  “It’s nice to know what options you have, and that this is indicated on the hand-out sheet. This way I know wat my possibilities are, and on which chapter of the decision aid I should pay closer attention to.” | “I think it’s good that the hand-out sheet contains a graph that you can use to communicate the situation to the patient. They are often unaware of the severity of the situation, and when they have to start treatment.”  “I think it’s nice that you can personalize this sheet by drawing the situation of the patient in the graph. I don’t know if all my colleagues will do this, a lot of them just show a graph on the computer.” |
| Educational content | “You want information about your situation and your possibilities. It’s all in the decision aid. Better too much information than too little.”  “It is a lot, but it is all necessary to know to make this decision. The information is structured logically: first the basic information, and then the considerations. The tables are clear and I really like the educational videos.”  “I am already an expert by experience, but this seems like a good thing to have if you are a new patient. You get so much information in the beginning; with this you can process everything step by step.” | “I think patients need one place where they can view their options and have everything explained to them in laymen’s terms. This decision aid provides just that.”  “The educational information we give now are all different leaflets, brochures or websites like nieren.nl. Here everything is built on one platform. I think that can really help patients.”  “I think the tables are very important for patients. Some find it difficult to imagine how these treatments might affect their life. Here the advantages and disadvantages are stated clearly and honestly.” |
| Values-clarification and preference-elicitation exercises | “The exercises are clear and helpful. At a certain point you have to have the guts to make the choice. It’s not that simple of course, but these exercises helped me visualize and structure my thoughts.”  “This decision aid guides you in making a choice. It was nice that the exercises immediately followed the educational text about each treatment modality.” | “I think it’s good to help patients along the way by confronting them with these exercises. We should always encourage patients to think about their situation, and how their treatment options might affect them”  “It’s important to me that patients know what they want, and why they want it. I think it’s good that it doesn’t push people in a certain direction, but rather helps them think about their situation.” |
| Outcome-information | “If you don’t get it here, you’re going to look it up on the internet anyway. On the internet you come across the strangest things. Here this information is concise, and I know that it is reliable.”  “I chose to not view the information about survival. I think you should keep it in the decision aid though. Everyone should decide for themselves whether they want to see it or not.” | “The information on survival is only shown when patients click on it. I think that it is important but confronting information. However, we have to be honest with our patients.”  “The information of the respective treatment options on different life-domains is illustrative and important. I think this provides patients with the knowledge they need to answer my questions with information I need to know.” |
| Personal summary sheet | “This summary sheet will certainly support conversations between patients and their doctors. It’s like a roadmap of subjects you need to discuss.”  “It provides an estimation of in which direction a patient is thinking. I think that doctors could definitely use this information during their consultations.” | “I think it’s good that you get information on these topics from your patients. This way you are well equipped to discuss their values and preferences.”  “I thought the summary sheet was clear. It also clearly indicates that you can download and print it. I even tried it and it works. I can see myself using this.” |

***Note: this is a translation from Dutch to English.**
